# Healthcare Professionals’ Experiences and Perspectives on Self-Management Education for Breast Cancer Survivors in Australian Primary Care: A Qualitative Study

**DOI:** 10.3390/nursrep16070254

**Published:** 2026-07-20

**Authors:** Meng-Yuan Li, Tao Wang, Daniel Terry, Haiying Wang, Isabella Zhao, Jing-Yu (Benjamin) Tan

**Affiliations:** 1 School of Nursing and Midwifery, University of Southern Queensland, Ipswich, QLD 4305, Australia; mengyuan.li@unisq.edu.au (M.-Y.L.); alison.wang@ecu.edu.au (T.W.); daniel.terry@unisq.edu.au (D.T.); emily.wang@unisq.edu.au (H.W.); 2 Faculty of Health, Charles Darwin University, Brisbane, QLD 4000, Australia; isabella.zhao@qut.edu.au; 3 Institute of Health Research, University of Southern Queensland, Springfield, QLD 4350, Australia; 4 School of Nursing and Midwifery, Edith Cowan University, Joondalup, WA 6027, Australia; 5 Institute of Health and Wellbeing, Federation University, Ballarat, VIC 3353, Australia; 6 Cancer and Palliative Care Outcomes Centre, Queensland University of Technology, Brisbane, QLD 4059, Australia; 7 School of Nursing and Midwifery, The University of Notre Dame Australia, Fremantle, WA 6160, Australia

**Keywords:** self-management education, breast cancer, primary care, healthcare professional, qualitative study

## Abstract

**Purpose**: Breast cancer has been recognised as a chronic condition, with survivors experiencing long-term physical and psychosocial effects following treatment. Supporting breast cancer survivors to self-manage their health can optimise health-related quality of life and reduce symptom burden. Self-management education (SME) has been advocated at the policy and programme level in Australian primary care, but its uptake is often limited. This study explores healthcare professionals’ (HCPs) experiences and perceptions of SME in primary care, aiming to identify factors influencing its implementation and to support survivorship care for breast cancer survivors in Australia. **Methods**: A qualitative descriptive approach was adopted involving in-depth semi-structured interviews. Convenience sampling was used to recruit HCPs across the three primary care clinics in the Greater Brisbane area. The interviews were conducted based on an interview guide. Each interview was audio-recorded and transcribed verbatim. Descriptive content analysis was used to analyse the data. **Results**: Fourteen participants were interviewed across three primary care settings, including five general practitioners and nine registered nurses. Three categories were synthesised from the data: (1) Meaning and role of SM/SME; (2) Suboptimal SME in current practice; (3) Perceived factors impacting SME delivery in practice. Each category had two to six subcategories for the participants. **Conclusions**: The findings highlighted the importance and perceived benefits of SME for breast cancer survivors and HCPs in Australian primary care. However, the integration of SME into primary care remained suboptimal due to a combination of systemic, organisational, and healthcare provider factors. Evidence-based resources, particularly guidelines and training, are essential to equip HCPs to effectively integrate SME into routine care for breast cancer survivors.

## 1. Introduction

Globally, breast cancer (BC) incidence is estimated to rise to over 3 million by 2040 [1,2]. The latest report from the Australian Institute of Health and Welfare (AIHW) [3] estimated that 21,000 BC cases were diagnosed among Australian women in 2024. Australia and New Zealand have among the lowest breast cancer mortality rates globally, particularly among premenopausal individuals. The 5-year survival rate for BC in Australia reached 92% in 2016–2020 due to improvements in early detection and better treatment [4]. BC is now regarded as a chronic disease [5], as many BC survivors continue to suffer from persistent treatment toxicities (e.g., hot flashes, bone loss, pain) and symptoms (e.g., fatigue, insomnia) for up to 14 years after diagnosis [6,7]. These adverse health effects vary according to age and treatment type, affecting individuals’ quality of life (QoL) of for years [8,9]. They can also hinder adherence to routine follow-up treatments and care, ultimately compromising long-term survival outcomes [10].

Traditional cancer care, typically led by oncologists and hospital-based teams, focuses predominantly on monitoring cancer recurrence, with less attention given to the long-term symptoms experienced by BC survivors [11]. Primary care, which serves as the first point of contact with the Australian health system, plays a crucial role in the post-treatment care of BC survivors, particularly in managing persistent symptoms [12]. The Australian Cancer Plan 2023–2033, released by Cancer Australia, indicates that cancer is a chronic disease requiring long-term management and highlights the prioritisation of survivorship care [13]. Moreover, cancer is included in the National Strategic Framework for Chronic Conditions, which encourages collaboration between General Practitioners (GPs) and Specialists to manage this chronic condition effectively [14]. A growing body of evidence has highlighted the potential of primary care clinicians, including nurse-led care, GP-led care, and shared care models, in improving symptom management and overall QoL for cancer survivors [15,16,17]. Shifting cancer care from traditional hospital-based models to primary care- and/or home-based models has been emphasised as an effective strategy to improve BC survivors’ self-care skills and ensure their long-term health needs are met [18].

Self-management (SM) refers to “the individual’s ability to manage the symptoms, treatment, physical and psychosocial consequences and lifestyle changes inherent in living with a chronic condition” [19] (p. 178). Specifically, SM after treatment may involve addressing physical symptoms, managing emotions, acquiring information, building relationships with healthcare providers, and adapting to new roles [18]. It represents a shift from patients as passive recipients of treatment to individuals who are partners in managing their health [20]. Thus, support for SM is receiving increasing attention in healthcare, with education identified as a key strategy for enhancing self-management [21,22]. In cancer care, self-management education (SME) has been described as an ongoing process that develops the knowledge, skills and confidence needed to manage the biological, physical and psychosocial impacts of cancer and/or its treatment [21,23]. Effective SME generally involves collaboration among patients, healthcare professionals (HCPs) (e.g., GPs and nurses), and the healthcare system.

Within the primary care context, HCPs are especially crucial in conducting comprehensive needs assessments, delivering SME to patients, and providing ongoing follow-up through general practice. These services are particularly important for cancer survivors, who often have diverse and long-term health needs. However, the implementation of SME in Australian general practice remains limited [24,25]. Despite the growing recognition of SME in cancer survivorship care [26,27,28], little is known about primary care HCPs’ experiences and perspectives on SME for cancer survivors. Previous studies have mainly focused on the views of cancer survivors or caregivers participating in SM interventions [26,29,30], while the views of HCPs involved in SME and its delivery remain underexplored. Understanding HCPs’ experiences, perceived barriers, enablers, and support needs may therefore inform strategies to improve SME delivery and promote its integration into Australian primary care. As such, the aim of this study was to explore HCPs’ experiences and perceptions of SME for BC survivors, as well as the factors influencing the implementation of SME in Australian primary care practice. It is anticipated that this study will highlight current SME practice, particularly in primary care, and inform strategies to better integrate SME into routine cancer care, ultimately meeting the needs of BC survivors within primary care settings.

## 2. Materials and Methods

### 2.1. Study Design

The current study is part of a larger project designed in accordance with the Medical Research Council Framework for Complex Interventions. The overall aim of the larger project is to develop and validate a primary care clinician-led supportive care programme to facilitate the SM of symptom distress among BC survivors. The project includes four study phases. In Phase I, two systematic reviews of randomised controlled trials [31] and clinical practice guidelines [32], along with an overview of systematic reviews [33], were conducted to identify evidence-based self-managed non-pharmacological interventions and implementation barriers and enablers in primary care. To further inform the development of a context-specific supportive care programme, this qualitative descriptive study was subsequently conducted to explore HCPs’ experiences and perceptions of SME, as well as factors (e.g., barriers and enablers) influencing its delivery in Australian primary care. A qualitative descriptive approach, informed by Sandelowski [34], was used because it enables a “comprehensive summary of an event in the everyday terms of those events” (p. 334) and is well suited to generating practice-relevant findings. This design is particularly appropriate when the aim is to explore HCPs’ experiences in healthcare settings while remaining close to participants’ accounts. The reporting of this study was guided by the Standards for Reporting Qualitative Research (SRQR) [35].

### 2.2. Participants and Recruitment Procedures

Convenience sampling was used to recruit eligible participants from three primary care clinics in the Greater Brisbane area, Queensland, Australia. The three clinics were community-based general practices located in different suburban areas of Greater Brisbane (not remote and rural areas) and provided general primary care services to the broader community. Specifically, following ethical approval, a member of the research team (I.Z.) approached the three primary care clinics and introduced the study to eligible staff, including the study aim, procedures, and the potential risks and benefits. Among clinicians who expressed interest, a participant information sheet was further provided to facilitate informed decision-making regarding participation. Written informed consent was obtained prior to data collection. Inclusion criteria encompassed participants being registered primary care clinicians, including GPs and registered nurses (RNs), who were willing to participate in the study; were practising in a primary care setting; and had a minimum of six months’ experience in the management of BC survivors.

### 2.3. Sample Size

Sample size was determined by data saturation, which was considered as the point at which no new meaning units or variations in participants’ experiences could be identified [36]. Data saturation was closely monitored throughout the data collection process. As sampling and data collection continued, the research team (I.Z., M.-Y.L., and T.W.) met regularly to assess whether new information emerged from the transcribed interviews. After fourteen interviews, no new insights or meanings were identified, confirming that saturation had been achieved. A joint decision was made to cease participant recruitment.

### 2.4. Data Collection

Data collection was conducted between September 2022 and November 2023. In-depth interviews were conducted via telephone or face-to-face, and were undertaken where the participants felt most comfortable, based on the participants’ preferences, or wherever was most convenient for them (e.g., the meeting room in their workplace). All interviews were audio-recorded. All interviews were conducted by the same member of the research team (I.Z.). Participants’ demographic data (e.g., age, gender, years of work experience) were also collected using a pre-designed basic information form.

### 2.5. Development of Interview Guide

The interview guide was developed comprehensively based on the expertise of the research team, previous qualitative and mixed-methods studies regarding HCPs’ experiences in SME [37,38,39]. The interview guide was further tested through a pilot interview, and minor wording changes were made to improve the clarity of the questions. The final interview guide included nine key questions.

The questions explored participants’ understanding and attitudes towards SM/SME for BC survivors in primary care (e.g., What does the term “self-management” mean to you?); participants’ experiences of delivering SME to BC survivors and their perceived effects/outcomes of delivering SME (e.g., Can you please tell me what self-management strategies are usually provided to breast cancer patients by you or your centre?); perceived factors impacting the delivery of SME in practice (e.g., In your opinion/based on your experience, what are the barriers to delivering self-management education or improving breast cancer patients’ self-management skills/knowledge?). Probing questions such as “Can you give some examples?”, “Can you describe the process you used in detail?”, and “Can you tell me why?”, were also used to elicit more detailed responses. The detailed interview guide is provided in the Supplementary Materials.

### 2.6. Data Analysis

All audio-recorded interviews were transcribed verbatim using Microsoft Word by one author (M.-Y.L.) and double-checked by another author (T.W.). Qualitative content analysis was used to analyse the data, which included four stages for category development: ‘initialization,’ ‘construction,’ ‘rectification,’ and ‘finalisation’ [40]. During the initialisation stage, two authors (M.-Y.L. and T.W.) read and re-read the transcripts to become familiar with the data. Relevant phrases, sentences, and/or paragraphs were highlighted using a line-by-line approach to identify meaning units and generate codes. Codes were developed inductively from the transcripts rather than from a pre-existing coding framework, with a focus on the explicit meanings conveyed by participants. During the construction stage, the preliminary codes were compared and grouped according to shared meanings and then organised into subcategories and categories aligned with the research objectives. During the rectification stage, the preliminary subcategories and categories were further refined by reviewing and reappraising all codes and the full dataset to ensure that they accurately reflected the original data. Lastly, during the finalisation stage, representative quotes were extracted from the transcripts to support each subcategory and/or category and to present the findings of the content analysis [40].

### 2.7. Rigour

Several strategies were used to enhance the rigour of this study. The research team had different academic, professional, and cultural backgrounds, which may have influenced the study design, data collection, and interpretation of the findings. The team included male and female researchers, Doctor of Philosophy (PhD) holders, and PhD students with backgrounds in health services research. Credibility was supported by encouraging participants to share both positive and negative experiences during the interview, double-checking all transcripts for accuracy, and involving two researchers (T.W. and M.-Y.L.) in data coding. Ten transcripts were initially coded by T.W., who is an experienced researcher in cancer care with extensive qualitative research experience, and reviewed by M.-Y.L., who then independently coded the remaining transcripts. Coding results were compared and discussed throughout the analysis by the two researchers, with peer review by a third researcher (J.-Y.T.) to detect any bias or inappropriate subjectivity in the authors’ interpretations of the data. The identified subcategories and categories were also reviewed by the entire research team. Dependability and confirmability were maintained through a systematic qualitative content analysis process, including initialisation, construction, rectification, and finalisation [40]. This included independent coding, reflexive discussion, team-based review, and the use of representative participant quotes to support the findings. Transferability was supported by providing detailed information on the study context, researchers’ backgrounds (e.g., their qualifications and research experience), participants’ characteristics, sampling method, data collection, and data analysis procedures, enabling readers to assess the applicability of the findings to other contexts.

As a researcher and Australian RN with previous work experience in primary care, the interviewer (I.Z.) had professional familiarity with the group and setting in this study. This familiarity facilitated recruitment through established professional networks and knowledge of the Australian primary care setting. It may also have helped build trust and reassure participants that the study was relevant to their daily clinical practice [41]. At the same time, the interviewer recognised that her professional background and prior experience could influence interactions with participants and the interpretation of their accounts. To minimise assumptions of shared understanding, participants were encouraged to explain and elaborate on their experiences in their own words. Reflexivity was ensured through the maintenance of reflective field notes by the interviewer (I.Z.), documenting reflections on contextual observations, participants’ perspectives, initial insights, and potential researcher influence during data collection.

## 3. Results

### 3.1. Characteristics of the Participants

Fourteen participants were interviewed, including nine registered nurses and five GPs. The sample was predominantly composed of nurses, reflecting common workforce demographics in primary care. The years of work experience among HCPs ranged from 3 to 20 years, providing perspectives across different career stages (Table 1). The mean interview length was 14.5 min (11–22 min).

### 3.2. Categories 

**Figure 1:**
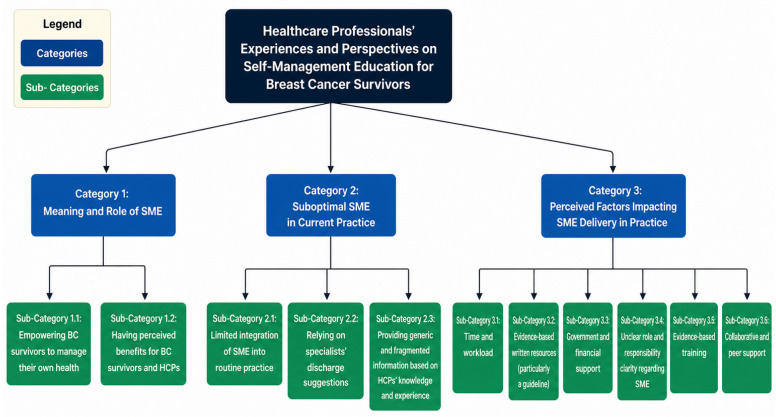
Conceptual map of categories and sub-Categories. Note: SM, self-management; SME, self-management education; BC, breast cancer; HCPs, healthcare professionals.

Content analysis identified three categories and 11 subcategories (Table 2). These interconnected categories and subcategories illuminate HCPs’ understanding of SM/SME, experiences in delivering SME, and factors influencing SME in general practice (Figure 1).

#### 3.2.1. Category 1: Meaning and Role of SM/SME

This finding demonstrates that HCPs hold clear and consistent views on SM/SME, recognising its importance for BC survivors. The benefits of SM/SME were acknowledged in various aspects, both for BC survivors and HCPs themselves. Within this category, three subcategories were evident:

Empowering BC survivors to manage their own health

Almost all HCPs demonstrated a good understanding of the meaning of SM for BC survivors. It was consistently described as empowering BC survivors to manage their health over the long term with ongoing support from HCPs. The challenges may include treatment toxicities, persistent symptoms, mental health concerns and other forms of distress that may emerge across the disease trajectory. The following definitions illustrate the dual role of SM as a way to enhance BC survivors’ skills and knowledge, and as a motivation to promote engagement among BC survivors.

*“So self-management could mean, um, patients coping with the effects of the treatment itself…as well as any associated commodities that come following the treatment or that they already have. So they would be able to manage it”*

(Participant 8)

*“so I think self-management is, uh, is really about how to empower or enable the patients to look after themselves. So that’s my understanding. Because in order to manage something, they have to know their conditions, they have to know, uh, resources and, you know, support. They have to look after themselves. So...just to help them to have the knowledge, uh, to know what to do”*

(Participant 5)

*“I think it’s more like, when patients they know what to do, like, they understand their conditions. They know when they need to seek help, and especially if they experienced some symptoms”*

(Participant 13)

However, one participant expressed a different view, stating that SM/SME may not mean much for cancer survivors when it comes to cancer progression. In situations where patient outcomes may be affected, a GP preferred to take over or refer patients to specialists, rather than encouraging SM. This viewpoint is perhaps unsurprising, as major clinical concerns (e.g., monitoring for recurrence or cancer progression) often take priority during the limited time available in general practice consultations.

*“I don’t really think the patients can do much (by themselves). Basically, sometimes if you worry about, you know, the cancer has like, develop or recur or change to a different part. There’s a need to come to the GP, just reset concern. If they worry about something that is not right. And GP will do some like relevant investigations. If this thing is necessary, I probably refer back to the specialists”*

(Participant 11)

Having perceived benefits for BC survivors and HCPs

In this study, most HCPs held a positive attitude toward SM/SME in cancer care. Effective SME was perceived to reduce unnecessary rehospitalisation and improve the overall health of BC survivors, which reflected HCPs’ confidence in their ability to provide SME and set goals collaboratively with BC survivors.

*“Proper self-management and having good knowledge of the condition in general would, I believe, help the patient. Um, again, keep them from going to hospitals unless necessary”*

(Participant 2)

*“That (patient education) can help them to improve their overall health”*

(Participant 13)

Given that patients commonly turn to internet-based resources for health-related information, SME would be more reliable and effective for BC survivors than information acquired from other resources.

*“Yes. self-management education resources...rather than Googling? Definitely that would be an answer...That I would be able to provide, um, to the patients”*

(Participant 10)

By enhancing patients’ confidence and competence in self-care, SME was perceived to reduce unnecessary use of health services. One GP believed that effective SME could reduce the demand for health-related resources by enabling patients to manage their health more independently.

*“You know, less resources needed from the doctors or the nurses if they’re able to take care of themselves”*

(Participant 14)

#### 3.2.2. Category 2: Suboptimal SME in Current Practice

Even though the potential benefits of SME were highlighted, the implementation of SME in general practice remained suboptimal. Both GPs and nurses consistently indicated that SME was not a main focus for BC survivors in routine care, with current practice heavily depending on specialists’ discharge recommendations. Only very limited, generic, and fragmented advice (e.g., lifestyle modifications, diet, or exercise) was provided based on the HCPs’ personal knowledge and experience.

Limited integration of SME into routine practice

All HCPs reported rarely integrating SME into routine practice for BC survivors, as the cancer management in Australia is primarily dependent on Specialists. Moreover, SME-related information was generally provided only when patients expressed specific concerns during their appointment. Verbal communication was the most common method of delivery.

*“For us it (SME) is very, very limited. So because like the breast cancer patients are mainly and the management with the specialist of the breast clinic of the hospital. So we don’t do anything in writing, but verbally”*

(Participant 3)

*“It’s just, um, providing them with information and hearing their concerns and dealing with that in every appointment that we have with them”*

(Participant 4)

Relying on specialists’ discharge suggestions

Cancer care in Australia is largely specialist-driven, with Specialists typically coordinating the patients’ follow-up plans. As such, they are seen as the primary source of authoritative guidance, including patient education.

*“Normally, uh, when the specialist would give us that information. But a certain patient has that. Then we follow whatever suggestion that they have”*

(Participant 12)

Some HCPs may view cancer-related education as outside the traditional scope of primary care, especially if they feel undertrained or inadequately supported in oncology-related topics. This further reinforces the dependence on Specialists’ inputs. When BC survivors presented with specific concerns or distress, they were usually referred to Specialists or other support groups for further intervention.

*“What my usual practice would be is refer to specialists and follow up as needed by the patient”*

(Participant 10)

*“And then some breast cancer support groups can sometimes be of help as well, so I can direct patients to approach those breast cancer support groups as well”*

(Participant 8)

Providing generic and fragmented information based on HCPs’ knowledge and experience

While SME is increasingly promoted, there is no widely implemented standardised SME programme for cancer survivors within Australian general practice. Therefore, nearly all HCPs described that they have limited experience in delivering SME to BC survivors. Their practice was largely based on personal knowledge and experience. The information provided was often fragmented and varied. Moreover, the content of SME tended to be generic and similar across providers, focusing on broad lifestyle modification, diet and exercise advice.

*“Just general advice or education, but it all depends on individual patient”*

(Participant 9)

*“Yeah, so, so these are really, I guess can call it generic, but these are very similar”*

(Participant 2)

*“It’s just from our experience and then from the past experience as well”*

(Participant 7)

#### 3.2.3. Category 3: Perceived Factors Impacting SME Delivery in Practice

When dedicated to implementing SME in general practice, HCPs identified significant barriers, including time constraints, workload burden, resource limitations, insufficient government and financial support, inadequate training and unclear role and responsibilities regarding SME. Many HCPs emphasised the urgent need for accessible, evidence-based written resources (particularly a guideline) to support consistent practice. Additionally, they highlighted the importance of evidence-based training, government and financial support, collaborative and peer support to facilitate SME integration into routine care.

Time and workload

Time and workload (13/14) were identified as one of the most critical factors affecting SME in practice. Most HCPs described being very busy in primary care settings, leaving them with insufficient time to provide meaningful SME for BC survivor. They had to cope with and manage other chronic diseases and acute situations other than the BC.

*“And also. You know, you are a GP doctor, as in primary care and every doctor will be very busy. And we don’t have enough time”*

(Participant 1)

*“Um, because we not only deal with cancer, we also deal with lots of chronic conditions. Also, acute stuff, like, wound dressings, sudden emergencies. So, we don’t really have time to sit down and (provide education)”*

(Participant 2)

Some of the HCPs expressed that providing SME would be more feasible if they had enough staff and time. They highlighted that a formal education session often requires considerable time:

*“Yeah. We don’t have enough time. We don’t have enough staff. If we have more time and more staff, I’m sure everyone can do better”*

(Participant 4)

*“So, because I guess to deliver such a good education session, it does… You can’t do that in five minutes. So, you probably need to allocate 20 min here”*

(Participant 13)

Evidence-based written resources (particularly a guideline)

Evidence-based resources, particularly guidelines, were another vital factor in enabling the implementation of SME in general practice (14/14). HCPs expressed frustration over the lack of structured support, stating that the absence of practical guidelines limited their ability to deliver consistent and formal education to BC survivors.

*“I wouldn’t really say we deliver anything, you know, formally. Yeah, because we don’t really have any guidelines to support us to do that”*

(Participant 5)

*“And then the most importantly, we need to have those guidelines and then reference, um, reference for the nursing staff to study. And then for…… unfortunately……, at the moment, at GP centre, we don’t have those guidelines”*

(Participant 7)

Some HCPs indicated that if scientific guidelines were available, it would effectively enhance SME delivery. Promoting these guidelines across medical centres to enhance awareness of HCPs on SME for BC survivors is also highlighted:

*“Like what I mean is like more obvious, like well-known, uh, guidelines, like simple ones to, for us to actually follow and then obviously to promote, to like a lot of like medical centres to know about this”*

(Participant 3)

*“Yeah, absolutely. Um, it’s, it’s just good if there are some scientific guidelines to help us as well and make sure that… each patient that we treat has standardised care and that we are not forgetting anything. Obviously, we try our best to give the best care we can”*

(Participant 6)

A specific and validated guideline is warranted in the future to ensure SME delivery effectively. In addition, evidence-based written resources (e.g., patient handouts and brochures) available in primary care settings would help HCPs deliver SME more effectively, allowing BC survivors to access information freely.

*“I just wanted to let everyone know breast cancer is very big part for women… So, we’re just hoping there’s more guidelines coming out shortly”*

(Participant 7)

*“… the patient handout can be very handy because even if I don’t have time, what if we can actually put the information in brochure, like somewhere outside in the waiting room, and then when they are waiting, they can actually grab it themselves. … And number two is like when they’re seeing us, if we don’t actually have enough time, we say, look, all the information that I want to tell you is actually in this little brochure, so go home and make sure you have a good read. And then if you do have any questions, come back to see us”*

(Participant 3)

Government and financial support

Half of the HCPs recommended that they require government and financial support to promote the delivery of SME in general practice. A key concern raised was that SME is not currently covered by Medicare or private health insurance, making it difficult to prioritise within time-limited consultations. Without dedicated funding and policy support, SME delivery was difficult to maintain, despite its recognised value in supporting BC survivors.

*“… if the government could provide, you know, specific funding, to help us initiate this kind of self-management education, that’ll be, that’ll be really, really good”*

(Participant 5)

*“Obviously it’s about education, about how they do it to themselves about the self-management, but when we actually spend the time with them, but there is no relevant like Medicare rebate”*

(Participant 3)

Despite the challenges, one HCP expressed optimism about future developments, stating that BC has been recognised as a chronic condition within the healthcare system. This shift has prompted government efforts (e.g., Medicare Benefits Schedule) to enhance support and funding for chronic disease management, which may positively impact the delivery of SME in general practice. The participant suggested that forthcoming changes could strengthen the role of primary care in supporting BC survivors.

*“And because breast cancer is also part of the chronic disease as well, and, uh, the government is, especially Medicare department is making some change at this stage and put more funding to the chronic disease and, uh, they are going to make some changes in the next couple years’ time. So…”*

(Participant 1)

Unclear role and responsibility clarity regarding SME

Several HCPs expressed uncertainty about whose responsibility it is to deliver SME to BC survivors. This uncertainty focused on whether the responsibility should lie with primary care clinicians or remain with specialists. The lack of clearly defined roles led to inconsistency in practice and confusion among HCPs, particularly among clinicians trained in different healthcare systems and countries.

*“I think probably the specialists will organize that for them. But if it’s not organized by a specialist, I think maybe GP will take the role to, to run that test as well”*

(Participant 11)

*“Because I come from the UK and we have services streamlined, we don’t really get involved because they realise how important GP time is. They don’t really expect GPs to educate patients because it’s not what we are set for”*

(Participant 10)

Evidence-based training

Some HCPs expressed concerns about insufficient training to address BC-specific issues, particularly in areas such as follow-up care and recognising when medical intervention is needed. Without appropriate, evidence-based training, they felt uncertain about their competence to support BC survivors through SME. This lack of training contributed to hesitation in delivering accurate and meaningful guidance.

*“Make one quite hesitant about providing proper advice are very mean. because, you know, having evidence-based training and information that we can give to patient”*

(Participant 2)

*“We don’t actually have the adequate training about like what shall we provide to (breast cancer patients), when we’re actually doing the (education), when we actually educate the patients who had like breast cancer treatment, and like what shall they look at and when shall they contact us, when and how often do we review them?”*

(Participant 3)

Collaborative and peer support

Delivering meaningful SME for BC survivors is complex, requiring a deep understanding of, and adaptation to each patient’s unique needs and preferences. Two HCPs advocated for a collaborative, multidisciplinary approach involving various professionals, such as GPs, nurses, specialists, dietitians and other allied health professionals. Such teamwork not only supports the development of SME plans but also facilitates better access to a range of health services.

*“Well, maybe getting, gathering all the information. From the GP, from the doctor, from the nurses, and get the results from the, from the dietician, get the results, and then from there, you create a plan of action”*

(Participant 12)

*Yes, it is. It (a team approach), it requires the detail, uh, from everyone else, all the, the allied health and everybody know”*

(Participant 12)

*“And, uh, so just involved and, uh, uh, more special doctors working together”*

(Participant 1)

Peer support among BC survivors was also seen as a valuable resource. The sharing of lived experiences and practical information could enable BC survivors to learn from each other. One participant highlighted that peer experiences could contribute to knowledge exchange that may also inform clinical practice.

*“Just chat with each other like, because obviously they had the same diagnosis, so they went through the, maybe, similar or same treatments, and then obviously they have a lot of information to share in between them”*

(Participant 3)

*“Because, I think, you know, there’s a lot of cases out there that we don’t know. And how they, uh, it could be good, maybe, to study our cases. What is the effective, what are the, the symptoms, more information out there. It could be a help also for patients and for the practice to learn”*

(Participant 12)

## 4. Discussion

This study explored HCPs’ experiences and perspectives on SME for BC survivors in Australian primary care. The findings reinforce the importance and benefits of SME, revealing consistent recognition among HCPs. However, despite supportive policy directions and generally positive attitudes among HCPs, SME remained inadequately integrated into general practice. Key barriers to SME delivery included workload burden, time constraints, and insufficient government and financial support. SME delivery for BC survivors was further challenged by limited evidence-based resources and training, as well as unclear roles and responsibilities regarding SME. These barriers suggest that SME delivery is primarily driven by a combination of systemic, organisational, and healthcare provider factors [42]. Evidence-based written resources, particularly specific guidelines, along with evidence-based training, and multidisciplinary and peer support, were perceived as key enablers of SME delivery in primary care.

HCPs viewed SME as an essential and beneficial approach to empowering BC survivors to manage their overall health. This perception aligns with policy directions on reinforcing the long-term self-management of cancer as a chronic disease in Australia [13]. Previous studies also emphasised the role of patient education in self-management and its importance in the care of cancer survivors [21]. The potential benefits of SME for healthcare resource utilisation (e.g., reduced readmissions) were also recognised in this study, consistent with findings from previous studies [21,43]. However, SME was suboptimally integrated into routine care for BC survivors in the primary care settings, suggesting a disconnect between policy expectations and routine clinical practice.

Key implementation experiences and strategies may be drawn from other chronic conditions, such as diabetes SME in Australian primary care. Although diabetes SME is strongly supported within the Chronic Care Model, its routine delivery in general practice during the initial implementation stage has been challenged by limited consultation time, variable patient health literacy, and insufficient multidisciplinary coordination [44]. Emerging evidence further suggests that structured care pathways, interprofessional collaborative practice, and redesigned delivery systems, such as nurse-led care coordination and proactive follow-up, may support better integration of SME into primary care [45,46]. While diabetes and breast cancer survivorship care differ, these experiences may offer thoughtful insights for considering how SME may be integrated into primary care-based BC survivorship support.

Developing a structured and cancer-specific framework to guide SME delivery in primary care practice may be one of the key strategies. The implementation of SME has largely relied on individual HCPs’ personal knowledge and experience, resulting in varied and fragmented practices rather than a standardised approach. One of the key barriers was the lack of a valid and reliable structured framework to guide SME delivery in practice. For example, in Australia, GPs may develop a Chronic Disease Management Plan [47] in consultation with patients diagnosed with diabetes. This plan identifies the patient’s health needs, outlines the services to be provided by GPs and other allied healthcare professionals (e.g., dietitians), and specifies the role of the patient in managing their condition. However, such structured care planning is not extended to cancer survivors in Australia. Although the Flinders Chronic Condition Management programme has been well established for clinical settings (e.g., general practice networks) and chronic conditions (e.g., cystic fibrosis), its application for cancer survivors remains limited [48]. A systematic review on support strategies (including SME) in primary care further emphasised the need for structured patient-provider exchanges, including one-on-one patient-provider consultations, ongoing follow-up and provision of self-care materials [49]. These findings also highlighted that a theoretical model (e.g., social learning theory) could provide a strong foundation for the effective delivery of SME [49].

Time and workload emerged as major factors within primary care consultations, limiting the opportunity for meaningful SME. This finding is consistent with previous research, which highlighted that structured and formal SME requires time and feedback from BC survivors [43,50]. The Australian health system relies heavily on its primary health care system [51], yet primary care HCPs are expected to address a wide range of health concerns, often within limited consultation times [52]. This structural feature of the primary care setting may partly explain why SME is often not prioritised over immediate clinical concerns such as recurrence monitoring and acute symptom management. Moreover, SME was often viewed as outside the perceived professional responsibilities and accountabilities of primary care HCPs. This perception contrasts with a key role of GPs as “promoting patient empowerment and self-management in regard to the co-benefits of improving individual and planetary health” [53]. While nurses, as autonomous practitioners supported by an organisational context, can promote the successful delivery of SME in general practice [50], current evidence indicates that SME should be delivered collaboratively rather than being assigned to a single professional ‘in charge’ [54]. Koczwara et al. (2021) emphasised that multidisciplinary collaboration plays a key role in delivering SME and improving patient outcomes throughout the cancer trajectory [54].

In general, the lack of clarity regarding roles and responsibilities remains a persistent issue in SME implementation [55]. Therefore, a key unresolved question is how SME can be delivered in a structured and scalable manner within primary care workflows. Given that existing chronic management models/programmes are generic rather than disease-specific, a specific guideline is warranted to support SME delivery in primary care for BC survivors. A good example is the application of the SME model in diabetes care [56], which demonstrates how a scientific guideline can clarify professional roles, enhance service quality, reduce healthcare costs and minimise practice variations [57]. Moreover, evidence-based written resources (e.g., patient brochures) could be an easy, time- and cost-efficient option for both BC survivors and healthcare practice settings. A valid patient brochure has demonstrated benefits in delivering SME for BC in general practice [58,59].

Another important barrier identified was insufficient government and financial support for SME implementation. In Australia, primary care funding is largely item-based through the Commonwealth Medicare Benefits Schedule (MBS), which is a comprehensive list of medical services, including consultations, procedures, and tests, subsidised by the Australian Government [60]. In response to the growing burden of chronic disease management in Australia, new MBS items or services related to chronic management have been added (e.g., coordination and planning) [25]. These new MBS items allow GPs to assign and claim time spent discussing and planning treatment for complex, long-term conditions. As such, further integration of SME for BC survivors into the MBS and the existing care models could help support and enhance the implementation of SME in primary care. Additionally, the need for evidence-based training was strongly emphasised to enhance the competence of HCPs to deliver effective SME. Many primary care HCPs lack the competence and confidence required to facilitate structured SM support programmes [61], particularly when comprehensive SME should encompass various aspects, including medication management, lifestyle modifications, mental health, social support, knowledge, information, and decision-making [62]. Rochfort et al. further indicated that specific training programmes on SME for HCPs could also contribute to patients’ self-care ability with chronic conditions [63].

### 4.1. Implications for Future SME Intervention

Based on the findings, several implications may be drawn for the development and delivery of future SME interventions. Future SME interventions may be embedded within structured and cancer-specific care pathways to support more consistent integration into routine chronic disease management. Such pathways may help clarify the roles of different HCPs, strengthen multidisciplinary collaboration, and ensure that SME is not delivered as an isolated or occasional activity. Intervention resources may also need to be standardised, evidence-based, and practical for use within time-constrained consultations. For example, patient brochures, structured education materials, or brief SME scripts may assist primary care providers to deliver more consistent advice while allowing some tailoring to survivors’ individual needs. Nurse-led follow-up and care coordination may represent a feasible approach to enhancing continuity of SME, monitoring ongoing concerns, reinforcing self-management strategies, and facilitating referral or escalation when required [64]. The findings further suggest that implementation strategies may need to address both provider-level capability and system-level feasibility. Targeted training for primary care HCPs could help build confidence in delivering cancer-specific SME. In addition, closer alignment between SME-related activities, existing Medicare funding mechanisms, and primary care workflows may be important to support future sustainability.

### 4.2. Limitations

This study has several limitations. Participants were recruited from primary care clinics in the Greater Brisbane area using convenience sampling, which may introduce selection bias and limit the transferability of the findings to regional and remote contexts, as well as to other Australian states and healthcare contexts. The sample was limited to GPs and nurses and may not reflect the views of other primary care HCPs involved in SME, such as dietitians. Although semi-structured interviews enabled focused exploration, they may have limited discussion of issues beyond the interview guide. Despite using a comprehensive interview guide and an experienced qualitative interviewer with primary care experience, the relatively short interviews may have limited data depth, although this may also reflect participants’ limited experience with SME and its suboptimal integration into routine primary care. Although questions about BC survivors with comorbid conditions were included in the interview guide, insufficient data were generated to explore this issue in depth. The interviews also did not explicitly explore whether participants conceptualised SME and self-management support as distinct or integrated components of care. This study was thus unable to examine this conceptual distinction in depth, and future research could consider investigating how HCPs and cancer survivors understand and experience the relationship between education and support in self-management care. In addition, this study captured only HCP perspectives and did not explore BC survivors’ readiness, capacity, or preferences for SME. Future research should include survivors’ views to better inform the design and implementation of SME in primary care. Finally, although strategies were used to enhance analytical rigour, data interpretation may have been shaped by the research team’s prior experience in intervention development and implementation science, which may have led them to interpret participants’ insights through these lenses, for example, by focusing on barriers, enablers, feasibility, acceptability, and sustainability.

## 5. Conclusions

This study provides valuable insights into HCPs’ experiences and perspectives on SME for BC survivors in Australian primary care, highlighting its critical role in promoting long-term self-management. While the importance of SME and its perceived benefits were widely recognised, its implementation in primary care remains suboptimal. This study emphasised the need for specific guidelines to standardise SME delivery in general practice. Additionally, evidence-based resources and training are essential to equip HCPs with the required skills and knowledge to deliver meaningful SME. Future efforts should strengthen access to allied health services and ensure holistic support, including financial support, to enable primary care settings to effectively integrate SME into routine care.

## Figures and Tables

**Table 1:** Demographic characteristics of participants (*n* = 14).

Participants	Occupation	Gender	Age	Interview Place	Years of General Practice Experience
P1	General practitioner	Male	41	Face-to-face	11
P2	Registered nurse	Male	33	Telephone	7
P3	Registered nurse	Female	33	Telephone	10
P4	Registered nurse	Female	28	Face-to-face	4
P5	Registered nurse	Female	25	Face-to-face	3
P6	Registered nurse	Female	40	Face-to-face	12
P7	Registered nurse	Female	36	Face-to-face	14
P8	General practitioner	Male	45	Face-to-face	14
P9	Registered nurse	Female	54	Face-to-face	5
P10	General practitioner	Female	41	Face-to-face	12
P11	General practitioner	Male	58	Face-to-face	20
P12	Registered nurse	Male	58	Face-to-face	4
P13	Registered nurse	Female	30	Face-to-face	6
P14	General practitioner	Female	58	Face-to-face	>10 (unspecified)

Note: P, participant.

**Table 2:** Summary of the categories and subcategories.

Categories	Sub-Categories
Meaning and role of SME	Empowering BC survivors to manage their own health
	Having perceived benefits for BC survivors and HCPs
Suboptimal SME in current practice	Limited integration of SME into routine practice
	Relying on specialists’ discharge suggestions
	Providing generic and fragmented information based on HCPs’ knowledge and experience
Perceived factors impacting SME delivery in practice	Time and workload
	Evidence-based written resources (particularly a guideline)
	Government and financial support
	Unclear role and responsibility clarity regarding SME
	Evidence-based training
	Collaborative and peer support

Note: SM, self-management; SME, self-management education; BC, breast cancer; HCPs, healthcare professionals.

## Data Availability

The data presented in this study are available upon reasonable request from the corresponding author.

## Supplementary Materials

The following supporting information can be downloaded at: https://www.mdpi.com/article/10.3390/nursrep16070254/s1, Interview Guide for healthcare professionals.

## Public Involvement Statement

No public involvement in any aspect of this research.

## Guidelines and Standards Statement

The study reporting followed the Standards for Reporting Qualitative Research (SRQR).

## Abbreviations

The following abbreviations are used in this manuscript:

HCPs	Healthcare professionals
SME	Self-management education
BC	Breast cancer
GPs	General Practitioners
QoL	Quality of life
RNs	Registered nurse
