# Supplementary material for: Healthcare Professionals’ Experiences and Perspectives on Self-Management Education for Breast Cancer Survivors in Australian Primary Care: A Qualitative Study
Source: Nurs Rep. 2026 Jul 20;16(7):254. doi: 10.3390/nursrep16070254 (PMC13416165; doi:10.3390/nursrep16070254)
Supplement: Supplementary file 1 [file nursrep-16-00254-s001.zip › nursrep-4349317-supplementary.pdf]

Interview Schedule/Guide (Healthcare professionals)

Basic information:

**Interview Date**		**Interview place**		**Interviewee' Code**	
**Occupation**	☐ **GP**<br>☐ **RN**<br>☐ **Others (**please specify)<u>:__</u>	**Age:**		**Gender:**	
**Years of working experience**		**Roles at the GP center**			

Interview guide:

Section 1 – Introductory question:
Shall we start by finding out some information about you?
What are your primary responsibilities or roles within this GP centre?

Section 2 – Transitional question

During your routine practice, do you have any experience in delivering self-management education and support to breast cancer patients, either verbally or in writing?

Section 3– Key questions about self-management education and support

1. What does the term "self-management" mean to you?

2. In your opinion, what does self-management mean to breast cancer patients at the follow-up stage (who are receiving primary care after the anticancer treatment)?

3. Can you please tell me what self-management strategies are usually provided to breast cancer patients by you/ by your centre?
   (a) How did you choose/determine the specific strategies?
   (b) Did you refer to any guidelines/recommendations from relevant professional associations when providing self-management advice? Can you give some examples?
   (c) Have you provided lifestyle modification strategies/advice to breast cancer patients? Can you give some examples?
   (d) Have you provided symptom management strategies/advice to breast cancer patients? Can you give some examples?

4. Can you please tell me what strategies/approaches you usually use to deliver self-management education and support to breast cancer patients?
   (a) What kind of materials do you provide to patients?
   (b) Which format do you use to provide the material to patients?
   (c) When/how often do you provide self-management education/support to breast cancer patients?
   (d) What's your role in the process?

5. Can you please recall when you last delivered a self-management education/support session to a patient with breast cancer?
   (a) Can you describe the process you used in detail?

6. Do you think self-management education and support are effective for patients with breast cancer?
   (a) Can you tell me why?
   (b) How did you usually evaluate the effects of the self-management education and support you provided?

7. In your opinion/based on your experience, what are the **enablers** to delivering self-management education or improving breast cancer patients' self-management skills/ knowledge?

   (a) How do you think the enablers facilitated the delivery of self-management education?

8. In your opinion/based on your experience, what are the **barriers** to delivering self-management education or improving breast cancer patients' self-management skills/ knowledge?
   (a) How do the barriers affect the implementation of self-management education?
   (b) How did you solve the barriers you met?
   (c) What kind of support do you need to address the barriers you met?

9. Do you think self-management education/support are necessary for breast cancer patients with other chronic health conditions? Such as Cardiovascular diseases.
   (a) Can you tell me why?

Section 4– Concluding question
Is there anything else you would like to tell me about delivering self-management education and support to patients with breast cancer?

Thank you for your time today.
